# Supplementary material for: Genetic and environmental determinants of variation in the plasma lipidome of older Australian twins
Source: eLife. 2020 Jul 22;9:e58954. doi: 10.7554/eLife.58954 (PMC7394543; doi:10.7554/eLife.58954)
Supplement: Supplementary file 1. [file elife-58954-supp1.docx]

**Supplementary File 1A. Heritable Lipid Species**

| *Trait* | *h^2^ (95% C.I.)* | *h^2^_c_ (95% C.I.)* | *h^2^_E_ (95% C.I.)* | *ICC MZ (95% C.I.)* | *ICC DZ (95% C.I.)* | *p-CE* | *p-AE* | *p-E* |
| --- | --- | --- | --- | --- | --- | --- | --- | --- |
| Cer(d16:1_24:1) | 0.53 [0.22, 0.68] | 3.03E-14 [0.00, 0.23] | 0.47 [0.32, 0.66] | 0.53 [0.34, 0.68] | 0.27 [0.17, 0.38] | 0.005 | 1.00 | 8.30E-06 |
| Cer(d17:1_24:1) | 0.59 [0.28, 0.72] | 1.42E-15 [0.00, 0.24] | 0.41 [0.28, 0.58] | 0.59 [0.42, 0.72] | 0.29 [0.21, 0.42] | 0.002 | 1.00 | 1.97E-07 |
| Cer(d18:0_22:0) | 0.51 [0.18, 0.66] | 4.38E-16 [0.00, 0.26] | 0.49 [0.34, 0.67] | 0.51 [0.33, 0.66] | 0.26 [0.16, 0.39] | 0.008 | 1.00 | 7.05E-06 |
| Cer(d18:0_24:0) | 0.43 [0.01, 0.59] | 9.96E-17 [0.00, 0.32] | 0.57 [0.41, 0.76] | 0.43 [0.24, 0.59] | 0.22 [0.12, 0.37] | 0.045 | 1.00 | 2.62E-04 |
| Cer(d18:0_24:1) | 0.44 [0.16, 0.60] | 2.39E-15 [0.00, 0.00] | 0.56 [0.40, 0.76] | 0.44 [0.24, 0.60] | 0.22 [0.12, 0.33] | 0.009 | 1.00 | 2.68E-04 |
| Cer(d18:1_23:0) | 0.49 [0.00, 0.64] | 1.34E-13 [0.00, 0.38] | 0.51 [0.36, 0.70] | 0.49 [0.30, 0.64] | 0.25 [0.15, 0.43] | 0.049 | 1.00 | 1.95E-05 |
| Cer(d18:1_24:1) | 0.55 [0.20, 0.68] | 5.79E-15 [0.00, 0.28] | 0.45 [0.32, 0.63] | 0.55 [0.37, 0.68] | 0.27 [0.19, 0.41] | 0.006 | 1.00 | 8.41E-07 |
| Cer(d18:1_25:1) | 0.55 [0.07, 0.68] | 1.72E-14 [0.00, 0.38] | 0.45 [0.32, 0.63] | 0.55 [0.37, 0.68] | 0.27 [0.19, 0.46] | 0.025 | 1.00 | 6.58E-07 |
| Cer(d18:2_24:1) | 0.53 [0.17, 0.67] | 9.47E-16 [0.00, 0.28] | 0.47 [0.33, 0.65] | 0.53 [0.35, 0.67] | 0.27 [0.18, 0.41] | 0.009 | 1.00 | 2.97E-06 |
| DG(18:0_18:1) | 0.54 [0.10, 0.67] | 1.34E-14 [0.00, 0.38] | 0.46 [0.33, 0.62] | 0.54 [0.38, 0.67] | 0.27 [0.19, 0.46] | 0.019 | 1.00 | 1.15E-07 |
| DG(18:1_18:1) | 0.46 [0.04, 0.61] | 1.37E-14 [0.00, 0.35] | 0.54 [0.39, 0.72] | 0.46 [0.28, 0.61] | 0.23 [0.14, 0.40] | 0.034 | 1.00 | 1.74E-05 |
| DG(18:1_18:2) | 0.42 [0.12, 0.58] | 1.21E-14 [0.00, 0.23] | 0.58 [0.42, 0.77] | 0.42 [0.23, 0.58] | 0.21 [0.12, 0.34] | 0.014 | 1.00 | 1.76E-04 |
| PC(37:3) | 0.41 [0.13, 0.58] | 6.30E-15 [0.00, 0.20] | 0.59 [0.42, 0.78] | 0.41 [0.22, 0.58] | 0.21 [0.11, 0.32] | 0.012 | 1.00 | 4.21E-04 |
| PC(42:4e) | 0.37 [0.05, 0.55] | 1.65E-17 [0.00, 0.22] | 0.63 [0.45, 0.84] | 0.37 [0.16, 0.55] | 0.18 [0.08, 0.30] | 0.030 | 1.00 | 3.70E-03 |
| PE(18:0p_22:6) | 0.33 [0.00, 0.51] | 2.02E-14 [0.00, 0.23] | 0.67 [0.49, 0.88] | 0.33 [0.12, 0.51] | 0.16 [0.06, 0.29] | 0.048 | 1.00 | 1.16E-02 |
| TG(15:0_18:1_22:6) | 0.39 [0.10, 0.55] | 9.94E-17 [0.00, 0.22] | 0.61 [0.45, 0.80] | 0.39 [0.20, 0.55] | 0.20 [0.10, 0.31] | 0.016 | 1.00 | 5.00E-04 |
| TG(16:0_16:0_24:0) | 0.39 [0.12, 0.56] | 1.18E-16 [0.00, 0.19] | 0.61 [0.44, 0.81] | 0.39 [0.19, 0.56] | 0.20 [0.10, 0.31] | 0.012 | 1.00 | 8.63E-04 |
| TG(16:0_16:0_24:1) | 0.44 [0.03, 0.60] | 3.07E-13 [0.00, 0.33] | 0.56 [0.40, 0.74] | 0.44 [0.26, 0.60] | 0.22 [0.13, 0.38] | 0.039 | 1.00 | 9.41E-05 |
| TG(16:0_18:1_23:1) | 0.35 [0.00, 0.51] | 3.34E-14 [0.00, 0.27] | 0.65 [0.49, 0.84] | 0.35 [0.16, 0.51] | 0.17 [0.08, 0.31] | 0.048 | 1.00 | 2.42E-03 |
| TG(18:0_17:0_18:0) | 0.29 [0.01, 0.47] | 1.15E-14 [0.00, 0.20] | 0.71 [0.53, 0.91] | 0.29 [0.09, 0.47] | 0.14 [0.04, 0.26] | 0.044 | 1.00 | 2.03E-02 |
| TG(18:0_18:0_18:0) | 0.43 [0.14, 0.59] | 8.76E-15 [0.00, 0.21] | 0.57 [0.41, 0.77] | 0.43 [0.23, 0.59] | 0.21 [0.12, 0.33] | 0.010 | 1.00 | 1.95E-04 |
| TG(18:1_18:1_23:1) | 0.42 [0.10, 0.57] | 4.31E-15 [0.00, 0.26] | 0.58 [0.43, 0.77] | 0.42 [0.23, 0.57] | 0.21 [0.12, 0.34] | 0.019 | 1.00 | 1.55E-04 |
| TG(18:1_18:1_24:1) | 0.37 [0.09, 0.54] | 1.02E-15 [0.00, 1.00] | 0.63 [0.46, 0.83] | 0.37 [0.17, 0.54] | 0.19 [0.09, 0.30] | 0.019 | 1.00 | 1.76E-03 |
| TG(18:2_17:1_18:2) | 0.40 [0.16, 0.57] | 1.54E-15 [0.00, 0.17] | 0.60 [0.43, 0.79] | 0.40 [0.21, 0.57] | 0.20 [0.10, 0.30] | 0.007 | 1.00 | 4.35E-04 |
| TG(19:1_18:1_18:1) | 0.49 [0.17, 0.64] | 1.29E-14 [0.00, 0.26] | 0.51 [0.36, 0.68] | 0.49 [0.32, 0.64] | 0.25 [0.16, 0.38] | 0.009 | 1.00 | 4.53E-06 |
| TG(19:1_18:1_18:2) | 0.40 [0.07, 0.56] | 2.48E-15 [0.00, 0.25] | 0.60 [0.44, 0.80] | 0.40 [0.20, 0.56] | 0.20 [0.10, 0.33] | 0.026 | 1.00 | 5.68E-04 |
| TG(25:0_16:0_18:1) | 0.45 [0.07, 0.60] | 4.17E-16 [0.00, 0.30] | 0.55 [0.40, 0.73] | 0.45 [0.27, 0.60] | 0.23 [0.13, 0.38] | 0.025 | 1.00 | 5.31E-05 |

Standardized additive genetic (*h^2^*=heritability), shared environment (*h^2^_c_*) and unique environment (*h^2^_E_*) variance components (95% CI) of lipids were obtained using the ACE model. The columns p-AE, p-CE, and p-E, respectively, denote the p-values from the likelihood ratio test comparing ACE model vs AE, CE, and E models. p-CE is also the p value for heritability because testing the component A=0 is equivalent to testing that the heritability is zero. C.I. indicates confidence interval; DZ, dizygotic; ICC, intraclass correlation coefficient; MZ, monozygotic.

**Supplementary File 1B: Heritability of summed lipid groups**

| Trait | A (95% C.I.) | C (95% C.I.) | E (95% C.I.) | ICC MZ (95% C.I.) | ICC DZ (95% C.I.) | p-CE | p-AE | p-E |
| --- | --- | --- | --- | --- | --- | --- | --- | --- |
| Cer | 0.56 (0.19, 0.69) | 5.58E-14 (0.00, 0.29) | 0.44 (0.31, 0.61) | 0.56 (0.39, 0.69) | 0.28 (0.19, 0.43) | 0.007 | 1.00 | 5.56E-07 |
| Cer(d18:1/X) | 0.56 (0.16, 0.69) | 3.63E-14 (0.00, 0.31) | 0.44 (0.31, 0.62) | 0.56 (0.38, 0.69) | 0.28 (0.19, 0.43) | 0.010 | 1.00 | 6.34E-07 |
| Monounsaturated SM | 0.51 (0.07, 0.65) | 4.99E-15 (0.00, 0.35) | 0.49 (0.35, 0.67) | 0.51 (0.33, 0.65) | 0.26 (0.17, 0.42) | 0.027 | 1.00 | 6.36E-06 |
| Triglyceride | 0.48 (0.18, 0.62) | 1.07E-15 (0.00, 0.24) | 0.52 (0.38, 0.70) | 0.48 (0.30, 0.62) | 0.24 (0.15, 0.36) | 0.007 | 1.00 | 8.58E-06 |
| Polyunsaturated TG | 0.48 (0.03, 0.62) | 9.12E-14 (0.00, 0.37) | 0.52 (0.38, 0.69) | 0.48 (0.31, 0.62) | 0.24 (0.15, 0.42) | 0.036 | 1.00 | 5.40E-06 |
| PE | 0.46 (0.10, 0.62) | 9.96E-16 (0.00, 0.28) | 0.54 (0.38, 0.73) | 0.46 (0.27, 0.62) | 0.23 (0.14, 0.37) | 0.020 | 1.00 | 6.81E-05 |
| Cholesterol | 0.43 (0.07, 0.59) | 3.59E-15 (0.00, 0.26) | 0.57 (0.41, 0.78) | 0.43 (0.22, 0.59) | 0.21 (0.11, 0.34) | 0.025 | 1.00 | 4.97E-04 |
| HDL-C | 0.42 (0.03, 0.77) | 2.70E-01 (0.00, 0.61) | 0.31 (0.22, 0.44) | 0.69 (0.56, 0.78) | 0.48 (0.30, 0.65) | 0.035 | 0.24 | 8.00E-15 |
| CL_TG62 | 0.42 (0.05, 0.57) | 1.80E-14 (0.00, 0.30) | 0.58 (0.43, 0.77) | 0.42 (0.23, 0.57) | 0.21 (0.12, 0.36) | 0.032 | 1.00 | 1.60E-04 |
| LDL-C | 0.40 (0.12, 0.57) | 2.00E-17 (0.00, 0.20) | 0.60 (0.43, 0.80) | 0.40 (0.20, 0.57) | 0.20 (0.10, 0.31) | 0.013 | 1.00 | 8.73E-04 |
| CL_TG49 | 0.39 (0.00, 0.55) | 2.22E-16 (0.00, 0.32) | 0.61 (0.45, 0.80) | 0.39 (0.20, 0.55) | 0.19 (0.10, 0.36) | 0.048 | 1.00 | 3.81E-04 |

Standardized additive genetic (A=heritability), shared environment (C) and unique environment (E) variance components (95% CI) of lipids were obtained using the ACE model. The columns p-AE, p-CE, and p-E, respectively, denote the p-values from the likelihood ratio test comparing ACE model vs AE, CE, and E models. p-CE is also the p value for heritability because testing the component A=0 is equivalent to testing that the heritability is zero. C.I. indicates confidence interval; DZ, dizygotic; ICC, intraclass correlation coefficient; MZ, monozygotic. CL_TG49 and CL_TG62 represent sum of triglycerides with 44-49 total carbons, and 56-62 total carbons respectively while Cer(d18:1/X) represents sum of all ceramides with an 18:1 acyl chain in the sn-1 position .
