# Supplementary material for: Genetic and environmental determinants of variation in the plasma lipidome of older Australian twins
Source: eLife. 2020 Jul 22;9:e58954. doi: 10.7554/eLife.58954 (PMC7394543; doi:10.7554/eLife.58954)
Supplement: Supplementary file 3. [file elife-58954-supp3.docx]

**Supplementary File 3**

Lipid (triglyceride)-gene expression associations listed by heritability and degree of saturation

|  | Heritable | Non heritable |
| --- | --- | --- |
| Saturated TG | *HDC*  *ADAM8, APMAP, CPA3, RP11-179G5.1, RPL4P2* | *APMAP, CPA3, HDC, MGAM*  *GATA2, KRT23* |
| Mono-unsaturated TG | *CPA3, LILRA6, LILRB3, SNHG1, TRIM51*  *GAB2, HDC, PRKCD,*  *RP11-350G8.3, RPSA, RPSAP15, RPSAP58, SNORA62* | *HDC*  *CPA3, GATA2, SLC45A3* |
| Polyunsaturated TG | *HDC*  *VAMP8*  *CPA3, GAB2, LILRA6, LILRB3, PRKCD, REPS2, RP11-179G5.1, RPL35P5, RPS10P14* | *HDC*  *CPA3*  *SLC45A3* |

This table includes gene transcripts associated with ≥3 lipids in each saturation class, and also includes the transcripts associated with the third highest number of lipids among the polyunsaturated TG class.

Abbreviations of gene names are based on Gene Ontology nomenclature:

ADAM8, A disintegrin and metalloproteinase domain-containing protein 8; APMAP, Adipocyte plasma membrane-associated protein; CPA3, carboxypeptidase A3; FPR1, Formyl peptide receptor 1; GAB2, GRB2-associated-binding protein 2; GATA2, Endothelial transcription factor GATA-2; HDC, Histidine decarboxylase; KRT23; Keratin, type I cytoskeletal 23; LILRA6, Leukocyte immunoglobulin-like receptor subfamily A member 6; LILRB3Leukocyte immunoglobulin-like receptor subfamily B member 3; MGAM, Maltase-glucoamylase; PRKCD, Protein kinase C delta type; REPS2, RalBP1-associated Eps domain-containing protein 2; RP11.179G5.1, Ribosomal Protein SA Pseudogene 18; RP11.350G8.3, Ribosomal Protein SA Pseudogene 17; RPL35P5, Ribosomal Protein L35 Pseudogene 5; RPL4P2, Ribosomal Protein L4 Pseudogene 2; RPS10P14, Ribosomal Protein S10 Pseudogene 14; RPSA, 40S ribosomal protein SA; RPSAP15, Ribosomal Protein SA Pseudogene 15; RPSAP58, Ribosomal protein SA pseudogene 58; S100A11P1, S100 Calcium Binding Protein A11 Pseudogene 1; SLC45A3, Solute carrier family 45 member 3; SNHG1, Small Nucleolar RNA Host Gene 1; SNORA62, 40S ribosomal protein SA; TRIM51, Tripartite motif-containing 51; VAMP8, Vesicle-associated membrane protein 8.
